# Does Fundus Fluorescein Angiography Procedure Affect Ocular Pulse Amplitude?

**DOI:** 10.1155/2013/942972

**Published:** 2013-07-30

**Authors:** Gökhan Pekel, Semra Acer, Ramazan Yagci, Ebru Nevin Cetin, Mehmet Can Hiraali, Hüseyin Kaya

**Affiliations:** Ophthalmology Department, Pamukkale University, 20070 Denizli, Turkey

## Abstract

*Purpose*. This study examines the effects of fundus fluorescein angiography (FFA) procedure on ocular pulse amplitude (OPA) and intraocular pressure (IOP). *Materials and Methods*. Sixty eyes of 30 nonproliferative diabetic retinopathy patients (15 males, 15 females) were included in this cross-sectional case series. IOP and OPA were measured with the Pascal dynamic contour tonometer before and after 5 minutes of intravenous fluorescein dye injection. *Results*. Pre-FFA mean OPA value was 3.05 ± 1.36 mmHg and post-FFA mean OPA value was 2.93 ± 1.28 mmHg (*P* = 0.071). Pre-FFA mean IOP value was 17.97 ± 1.99 mmHg and post-FFA mean IOP value was 17.81 ± 2.22 mmHg (*P* = 0.407). *Conclusion*. Although both mean OPA and IOP values were decreased after FFA procedure, the difference was not statistically significant. This clinical trial is registered with Australian New Zealand Clinical Trials Registry number ACTRN12613000433707.

## 1. Introduction

Fundus fluorescein angiography (FFA) is a procedure for the examination of chorioretinal circulation. It can be used to confirm the diagnosis, monitor the progress of posterior segment diseases, and assess the efficacy of certain treatments like laser photocoagulation and intravitreal injections. Although FFA is a safe procedure, some serious complications like allergic skin rash and anaphylaxis could be seen rarely [1]. The effect of fluorescent molecule or FFA procedure on choroidal circulation is not known much.

The dynamic contour tonometry (DCT) is a noninvasive and direct intraocular pressure (IOP) measuring device and is considered to accurately measure the IOP independent of the corneal thickness or corneal elasticity [2]. It also provides a continuous examination of the IOP and measures ocular pulse amplitude (OPA) which is accepted as an indirect measurement of the choroidal blood flow [3].

The OPA is generally considered as the difference between systolic and diastolic values of the pulsatile IOP. It gives us an opinion about the choroidal blood flow corresponding with the heart pulse as a function of time [4]. Choroidal circulation has a vital role in ocular physiology and may be affected in various ocular and systemic diseases [5, 6].

FFA is an imaging procedure using fluorescence ability, and it has been thought to have little or no effect on ocular physiology. The rationale of this study was that fluorescein sodium passes freely through the choroidal vessels into the extravasal space and this would have an influence on choroidal blood flows, either by an osmotic effect or a hemorheological effect. Also, influence of fluorescein sodium on the erythrocyte aggregation was shown in the past literature, which might be related with alterations in retina-choroidal microcirculation [7]. Although it was not possible to examine the tissue pathology in this study, we wanted to reveal the effects of FFA procedure on eye in terms of IOP and OPA.

## 2. Materials and Method

This study was a prospective cross-sectional case series and involved 60 eyes of 30 patients. The study was conducted in accordance with the ethical standards of Declaration of Helsinki and was approved by the Institutional Ethical Committee.

### 2.1. Study Population

All of the eyes had nonproliferative diabetic retinopathy (NPDRP). The diagnosis of NPDRP was based on indirect retinoscopy and fundus fluorescein angiography (FFA). All of the subjects underwent an ophthalmic examination including visual acuity assessment, biomicroscopy, air-puff tonometer, indirect retinoscopy, pachymeter, and macular optical coherence tomography before their participation in the study. Exclusion criteria were any ocular surgery other than phacoemulsification, corneal irregularities, history of glaucoma, uveitis, and any other retinal disorders except NPDRP. Also, the patients who had nausea and vomiting after FFA were excluded, since these situations could affect the OPA and IOP. Patients had refractive errors between −1.50 and +1.50 diopters spherical equivalent.

### 2.2. Measurement Techniques

FFA measurements were done with Topcon TRC 501X angiography (Topcon Corporation, Tokyo, Japan). FFA procedure was done as follows. 5 mL 10% fluorescein sodium was injected rapidly from median antecubital vein or dorsal hand veins. After injection, serial retinal photographs were taken for 5 minutes. Between postinjection 5 and 10 minutes, IOP and OPA were measured. In order to separate the effect of rapid photography from fluorescein sodium injection, we had taken immediate postcolored retinal photography OPA and IOP measurements of 10 eyes before the dye injection.

Intraocular pressure (IOP) and ocular pulse amplitude (OPA) measurements were done with the Pascal dynamic contour tonometer (Pascal DCT, Swiss Microtechnology AG, Port, Switzerland). This is a slit-lamp biomicroscopy mounted, self-calibrating, 7 mm tip diameter, and 1.2 mm pressure sensor diameter device. One drop of Alcaine (proparacaine chloride 5 mg/mL; Alcon, Fort Worth, TX) was instilled just prior to IOP and OPA measurements. Measurements were performed within a 5-minute period. Three DCT measurements were taken in order to have one good-quality measurement. Only quality 1 and 2 measurements were taken into consideration. All OPA and IOP measurements were performed by the same investigator (GP) before and after 5 minutes from fluorescein dye injection.

Optical coherence tomography (OCT) measurements were done with spectral-domain OCT (Spectralis, Heidelberg, Germany). Only central macular thickness was assessed by OCT. Corneal pachymetry measurements were done with Nidek UP-1000 ultrasonic pachymeter and central corneal thickness was recorded. The pachymeter probe was placed on the center of the cornea and the mean of five readings was calculated.

### 2.3. Statistical Analysis

Statistical analysis was performed with SPSS for Windows statistical software version 17.0 (SPSS Inc. Chicago, IL, USA). All data are written as mean ± standard deviation (SD). Paired *t*-test between pre-FFA and post-FFA OPAs and IOPs were performed to evaluate the effect of FFA on OPA and IOP. The Wilcoxon signed-rank test was used to analyze pre- and postretinal photography OPA and IOP values of 10 eyes. Age and macular thickness were assessed for a relationship with pre-FFA and post-FFA OPA and IOP differences using univariate linear regression analysis. The effects of lens status and gender on pre-FFA and post-FFA OPA and IOP differences were analyzed with independent samples *t*-test.

## 3. Results

The study evaluated 30 nonproliferative diabetic retinopathy patients (15 males, 15 females). Bilateral eyes were recruited for all the patients. The distribution of eyes according to NPDRP severity was as follows: mild NPDRP in 10 eyes, moderate NPDRP in 38 eyes, and severe NPDRP in 12 eyes.

Pre-FFA mean OPA value was 3.05 ± 1.36 (range: 1.40–7.60, median: 2.60) mmHg and post-FFA mean OPA value was 2.93 ± 1.28 (range: 1.10–7.00, median: 2.60) mmHg (*P* = 0.071). Pre-FFA mean IOP value was 17.97 ± 1.99 (range: 12.80–23.40, median: 18.10) mmHg and post-FFA mean IOP value was 17.81 ± 2.22 (range: 12.20–24.10, median: 17.80) mmHg (*P* = 0.407). Best corrected visual acuity values evaluated with Snellen chart ranged between 0.1 and 0.9 in decimals (mean 0.54 ± 0.28).

In order to evaluate the individual effect of successive retinal photography, we recorded post-photography OPA and IOP values of only ten eyes. Pre-retinal photography mean OPA value was 2.70 ± 0.66 mmHg and postretinal photography mean OPA value was 2.63 ± 0.61 mmHg (*P* = 0.45). Pre-retinal photography mean IOP value was 18.36 ± 1.49 mmHg and postretinal photography mean IOP value was 18.28 ± 1.99 mmHg (*P* = 0.78).

No adverse effects like allergic skin rush or anaphylaxis occurred after intravenous fluorescein dye injection. Some demographic and clinical characteristics of the subjects studied, including mean age, gender, lens status, central corneal thickness, and central macular thickness, are shown in Table 1.

When considered alone, age (*P* = 0.017; *R* ^2^ = 0.094) showed statistically significant association with pre-FFA and post-FFA OPA difference, while macular thickness (*P* = 0.119; *R* ^2^ = 0.074) did not show significant association with OPA change.

Age (*P* = 0.001; *R* ^2^ = 0.166) showed statistically significant association with pre-FFA and post-FFA IOP difference, while macular thickness (*P* = 0.421; *R* ^2^ = 0.020) did not show significant association with IOP difference.

In males, mean OPA value increased 0.093 mmHg; whereas in females, mean OPA value decreased 0.330 mmHg after FFA (*P* = 0.001). In males, mean IOP value decreased by 0.027 mmHg and in females mean IOP value decreased by 0.317 mmHg (*P* = 0.468).

In phakic eyes, mean OPA value decreased by 0.058 mmHg and in pseudophakic eyes, mean OPA value decreased by 0.300 mmHg (*P* = 0.101). In phakic eyes, mean IOP value decreased by 0.10 mmHg and in pseudophakic eyes, mean IOP value decreased by 0.38 mmHg (*P* = 0.548).

## 4. Discussion

FFA uses intravenous fluorescein dye in order to image retinal and choroidal circulation. It has become the gold standard technique in revealing many of the retinal diseases [8]. Since fluorescein molecule is relatively small, in a normal eye, it remains within retinal vessels but freely passes the walls of choroidal vessels [8]. In a diseased retina like diabetic retinopathy, fluorescein dye also crosses retinal vessels. There are many fluorescein derivatives and fluorescein sodium is used in FFA. There is lack of comprehensive data about the effects of fluorescein sodium and FFA procedure on ocular tissues. In this study, we examined IOP and OPA only in diabetic retinopathy, although there are many other ocular tissues, functions, and disorders that may be affected by FFA procedure.

Possible factors related to FFA procedure that may affect ocular tissues and functions are not clear. But, the amount of injected fluid, the fluorescein leakage both in diseased retinal vessels and choroid, anxiety of patients related to the examination, the effect of sequential photograph taking, and/or the fluorescence ability of injected molecule might affect IOP and OPA. But more probable factors are the osmotic effects and hemorheological effects of fluorescein sodium. We did not have the opportunity to investigate individual contribution of these factors, but we take into consideration FFA procedure in general.

Sargento et al. in their study about sodium fluorescein influence on the hematological properties of diabetic patients, revealed that sodium fluorescein injection during retinography increased whole blood viscosity, erythrocyte elongation index, blood pH, carboxyhemoglobin, and methemoglobin levels and caused a sudden structural or functional reduction of red cell acetylcholinesterase activity [9]. All of these factors might have an effect on choroidal blood flow and hence ocular pulse amplitude.

Another possible mechanism about sodium fluorescein effect on OPA might be the osmotic effect, since Fluorescite 10% has an osmolality of 572–858 mOsm/kg and plasma has an osmolality of 285–295 mOsm/kg. It is known that small changes in osmolality can have a marked effect on water transfer [10]. However, it was reported that 30 minutes after sodium fluorescein injection, the increase in plasma osmolality was statistically insignificant [9].

In this study, although both mean OPA and IOP values were decreased after FFA procedure, the difference was not statistically significant. But FFA procedure affected males and females differently in the aspect of OPA. Mean OPA value of males increased, whereas mean OPA value of females decreased after FFA. Possible explanation about this result might be the effects of gender on FFA procedure and OPA, since gender differences in health issues could be seen.

In the literature, various factors affecting OPA were investigated. Some of the factors that decrease OPA are carotid artery stenosis, accommodation, glaucoma, scleral buckling surgery, regional orbital anesthesia, Graves' ophthalmopathy, panretinal photocoagulation, physical exercise, diurnal variation (lowest OPA at 1:00–3:00 pm), advanced retinitis pigmentosa, and exudative age-related macular degeneration [11–21]. Some factors that does not alter OPA are smoking, intravitreal bevacizumab injection, trabeculectomy, valsalva maneuver, and systemic blood pressure [3, 22–25]. Some factors that increase OPA are water drinking and high IOP [26, 27]. Since OPA is accepted as an indirect measurement of choroidal blood flow and fluorescein dye freely passes from choroidal vessels into the extravasal space, we thought that FFA might have an influence on OPA.

Centofanti et al. investigated influence of sex and hormonal status on choroidal blood flow [28]. They used ocular blood flow tonograph to measure OPA. They found that premenopausal females (age: 17–42 years) had significantly higher rates of OPA when compared with age-matched males, whereas postmenoposal females (age: >55 years) had similar OPA values with age-matched males. They concluded this result might be due to hormonal status (oestrogen) [28]. Our female study group was generally composed of postmenoposal females. In our study, the difference between males and females was not based on mean OPA value; it was due to the different influence of FFA procedure on OPA between genders. Although we could not explain the exact mechanism of this result, it is known that gender influences occurrence and outcome of some disorders [29]. Also, we measured that pre-FFA and post-FFA mean IOP difference was higher in females than in males. Since IOP affects OPA, this might also explain different OPA measurements between males and females.

Schmidt et al. examined OPA in diabetes mellitus and reported that choroidal circulation remained unaffected as diabetic retinopathy advanced [30]. Their study group was composed of diabetic patients in three subgroups: no diabetic retinopathy, NPDRP, and proliferative DRP. In our study, only NPDRP patients were included into the study in order to maintain better homogeneity. We also examined the effects of fluorescein leakage amount in terms of macular thickness and found that it had no significant effect on OPA and IOP.

There are some restrictions related to this study. For example, ideal timing of OPA and IOP measurements after FFA is difficult to determine. Since taking photographs is very important in the first five minutes, it was not appropriate to disturb the examination. But we quickly measured OPA and IOP after 5 minutes from fluorescein injection. We did not perform FFA on normal subjects, but in order to maintain standardization of the patients, only NPDRP patients were included in the study. One may say if there were an issue, 50 years and millions of fluorescein angiograms later, we would have learned of it. But, according to us, it is important to document these data about FFA for the first time in the literature.

In conclusion, FFA procedure does not affect OPA and IOP values. But, age showed significant association with pre-FFA and post-FFA OPA and IOP differences. Interestingly, mean OPA value of males increased, whereas mean OPA value of females decreased after FFA. More comprehensive studies including various ocular and systemic diseases and measurements at different FFA stages are needed to reveal more effects of FFA on OPA.

## Figures and Tables

**Table 1:** Demographic and clinical characteristics of patients studied.

Age (years ± SD)	
Mean	59.00 ± 7.37
Range	47–75
Sex (%)	
Male	15 (50%)
Female	15 (50%)
Lens status (%)	
Phakia	45 (75%)
Pseudophakia	15 (25%)
Central corneal thickness	
(*µ*m ± SD)	551.93 ± 30.97
Central macular thickness	
(*µ*m ± SD)	357.41 ± 109.03
